# Visit-to-visit ultrafiltration volume variability predicts all-cause mortality in patients receiving hemodialysis

**DOI:** 10.1080/0886022X.2023.2194439

**Published:** 2023-04-03

**Authors:** Quanchao Zhang, Ning Wang, Ling Nie, Caibao Lu, Hongwei Chen, Wenchang He, Moqi Li, Yiqin Wang, Jinghong Zhao, Jiachuan Xiong

**Affiliations:** Department of Nephrology, The Key Laboratory for the Prevention and Treatment of Chronic Kidney Disease of Chongqing, Chongqing Clinical Research Center of Kidney and Urology Diseases, Xinqiao Hospital, Army Medical University (Third Military Medical University), Chongqing, PR China

**Keywords:** Visit-to-visit, ultrafiltration volume, variability, mortality, hemodialysis

## Abstract

**Purpose:**

Little is known about the effect of visit-to-visit ultrafiltration volume (UV) variability on the outcome. In this study, we investigated the association between visit-to-visit UV variability and all-cause mortality in patients receiving hemodialysis (HD).

**Methods:**

We consecutively enrolled patients who received maintenance HD in our center from March 2015 to March 2021. UV variability was defined using standard deviation (UVSD) and coefficient of variation (UVCV) (standard deviation divided by the mean). The relationship between UV variability and all-cause mortality was assessed using univariate and multivariate Cox proportional hazard regression models. Receiver operating characteristic curves were used to evaluate the predictive abilities of UVSD and UVCV for short-term and long-term survival rates.

**Results:**

A total of 283 HD patients were included. The mean age was 57.54 years, and 53% were males. Follow-up was done for a median of 3.38 years (IQR 1.83–4.78). During the follow-up period, 73 patients died. Cox proportional hazards models indicated that UVSD and UVCV (higher versus lower) were positively associated with all-cause mortality (*p*=.003 and *p*<.001, respectively), while in multivariable-adjusted models, only higher UVCV remained significantly associated with all-cause mortality in patients receiving HD (HR 2.55 (95% CI 1.397–4.654), *p*=.002). Moreover, subgroup analyses showed that the predictive performance of UVCV was more accurate among older patients, males and patients with comorbidities.

**Conclusions:**

Visit-to-visit UV variability, especially UVCV, is a helpful indicator for predicting all-cause mortality in patients receiving HD, especially for older patients, males and those with comorbidities.

## Introduction

Hemodialysis (HD) remains the most used kidney replacement therapy worldwide, accounting for over two-thirds of all kidney replacement therapies [1]. Compared with the general population and even peritoneal dialysis counterparts, patients receiving HD have significantly higher mortality [2]. Although dialysis technology and services have vastly improved, the outcome of HD is still not optimistic [3]. Among the known causes of death for patients receiving HD, cardiovascular disease is the major cause of mortality and accounts for more than 50% [4]. Consequently, exploring the risk factors is extremely important for improving the survival rate of HD patients.

HD patients frequently experience unpleasant symptoms, such as postdialysis light-headedness, fatigue and worsening cramps during dialysis treatment [5]. This suggests that volume management is essential and a vital component of dialysis treatment for HD patients [4]. Volume overload results in peripheral edema, breathlessness-induced hypertension, left ventricular hypertrophy, and heart failure [6]. Extracellular volume overload is a critical contributor to the high risk of cardiovascular mortality in patients undergoing HD [7,8]. However, aggressive volume removal is associated with intradialytic hypotension (IDH), myocardial stunning, and greater mortality and morbidity [9]. Currently, there are no precise indicators of fluid volume, and there is uncertainty about how to achieve ideal volume, especially in extracellular water.

In current clinical practice, dry weight targeting is subjective and influenced by many factors [10]. The difficulty lies in assessing volume status and deciding how much fluid to remove during HD, namely, ultrafiltration volume (UV). Previous studies have shown that a higher ultrafiltration rate (defined as UV per unit time and weight) contributes to poorer prognosis in HD patients [11,12]. However, there is little evidence to support it as a good indicator for volume control [13]. It is worth noting that, to date, little is known about the effect of visit-to-visit UV variability on the outcome. Thus, we aim to assess the impact of visit-to-visit UV variability on all-cause mortality in patients receiving HD.

## Materials and methods

### Study subjects

**Figure 1.**
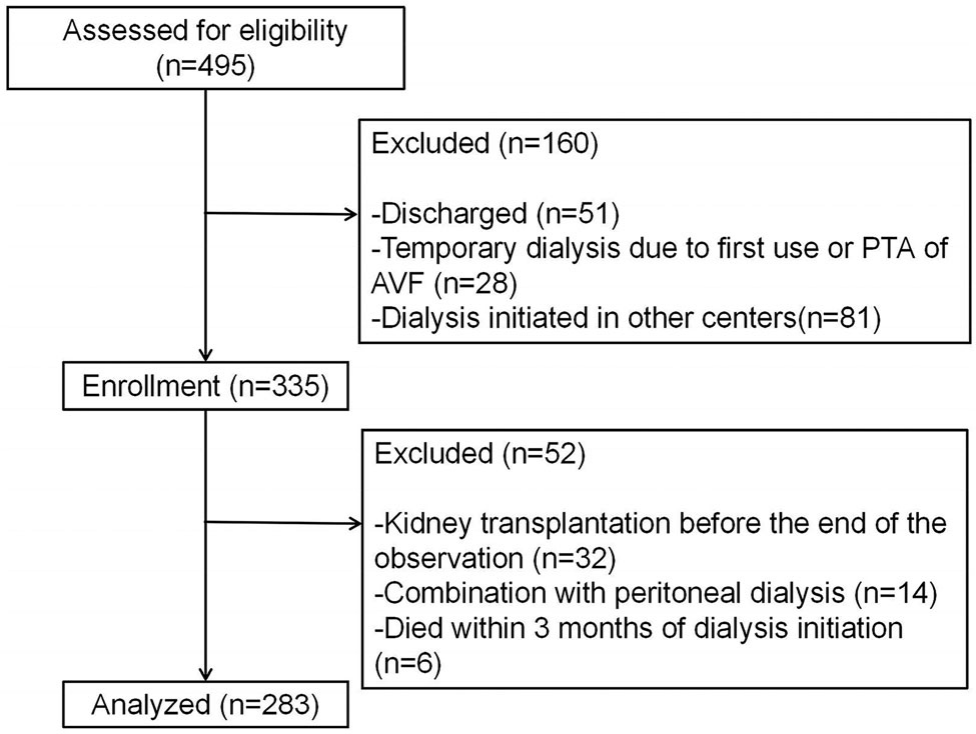
Flowchart showing the procedure for the selection of study participants. PTA: percutaneous transluminal angioplasty; AVF: arteriovenous fistula.

A total of 283 HD patients from the Department of Nephrology at Xinqiao Hospital, Army Medical University, from March 2015 to March 2021, were retrospectively analyzed (Figure 1). The inclusion criterion was as follows: patients received maintenance HD treatment in our center for at least 3 months. All patients received 4 h of conventional HD three times weekly. The sodium concentration in dialysis is usually 135–140 mmol/L, which should be selected according to blood pressure control. Individualized sodium concentration can be selected when hypertension is not well controlled, such as low-sodium dialysis, high-sodium dialysis, or sequential dialysis with sodium concentration from high to low. The follow-up period was from the first dialysis to the occurrence of the primary outcome. Otherwise, the final follow-up time was the most recent visit. The exclusion criteria were as follows: age below 18 years; received kidney transplantation in the follow-up period; combination with peritoneal dialysis; lack of clinical information data or clear medical history.

### Clinical and laboratory information

The following data were collected at the time of the first dialysis: age, sex, body mass index (BMI), B-type natriuretic peptide, presence of hypertension, diabetes, cardiovascular disease (myocardial infarction, cerebral infarction, cerebral hemorrhage, coronary heart disease, chronic heart failure, and peripheral vascular diseases), and chronic kidney disease etiology. Residual urine after 3 months of dialysis was collected during a 24-h period of the interdialytic interval. Intradialytic hypotension is defined as a decrease in systolic blood pressure by 20 mm Hg, which is based on K/DOQI clinical practice guidelines [14]. Blood pressure measurements were made pre- and post-HD every 30 min during the treatment. Hemoglobin, serum albumin, serum calcium, serum phosphorus, and serum intact parathyroid hormone (iPTH) were obtained from the most recent routine laboratory tests, in which blood samples were collected before HD.

### Definitions of UV variability

**Figure 2.**
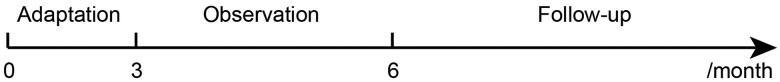
Schematic diagram of patient adaptation, visit-to-visit observation, and follow-up after dialysis.

After a 3-month dialysis adaptation period, a series of visits were conducted for six consecutive months from the observation period to evaluate UV variability. Every dialysis was regarded as a visit, and dialysis-related information was recorded. The patient’s adaptation, visit-to-visit observation and follow-up after dialysis are shown in Figure 2. The standard deviation (SD) and coefficient of variation (CV) were calculated using the indices of UV variability. CV is SD divided by mean. UV data during the observation period were recorded and used to calculate UV variability. The calculation formulas are as follows:

UVSD=∑i=1n(UVi−UV¯)2n−1 UVCV=UVSDmean

where *n* is the total dialysis time of each patient, and the mean was calculated from all UV data. For all included patients, the number of dialysis ranged from 129 to 1021, with a median of 562.

### Subgroup analysis

Patients were divided into different subgroups according to the following characteristics for further analysis: age greater than or less than 65 years, sex, presence or absence of diabetes, and presence or absence of CVD. Next, we analyzed the effect of UVSD and UVCV on all-cause mortality among different subgroup populations. The analysis method was the same as that for the whole population.

### Statistical analysis

Continuous variables are shown as the average ± SD or the median and interquartile range depending on the normal distribution results using the Kolmogorov–Smirnov test, whereas categorical variables are described as percentages (%). The *t*-test was applied for normally distributed data, and the Mann–Whitney *U*-test was applied for nonnormally distributed data. The Chi-squared test was applied for variable data. Hazard ratios (HRs) were estimated using Cox proportional hazard regression analyses for the relationships between all covariates (including demographic, clinical, and laboratory information) and all-cause mortality. The relationship between UV variability and all-cause mortality was plotted using the Kaplan–Meier method and assessed using the log-rank test. Receiver operating characteristic (ROC) curve analysis was used to compare the efficiency of two UV variability indices in predicting 6-month, 1-, 3-, and 5-year survival rates. The statistical significance of ROC was performed using DeLong’s test. All statistical analyses were performed with SPSS Statistics 23 (IBM Company, Chicago, IL). For multiple comparisons, Bonferroni’s correction was applied and *p*<.0125 was considered significant. In other cases, *p* value is set to .05.

## Results

### Baseline characteristics of the included patients

In the final analysis, 283 patients were included in the study. The average age of the participants was 59.5 years, with 53% of them being males. A total of 81 (29%) participants had diabetes, and 100 (35%) had cardiovascular disease. In 97% of cases, arteriovenous fistulas or arteriovenous grafts were used for dialysis. For CKD etiology, 114 (40%) patients had glomerulonephritis, 73 (26%) had diabetic nephropathy, 30 (11%) had hypertension, and 48 (17%) had other causes. A summary of the patients’ characteristics and laboratory data is provided in Table 1. The median value of UVSD was 627.6, while the median UVCV was 0.29. Based on these values, patients were divided into two groups (higher and lower). Compared with patients with lower UVSD, patients with higher UVSD were much older and more likely to be female but had significantly lower levels of serum phosphorus and iPTH. However, there was only a significant difference in BMI, dialysis vintage and IDH incidence between the higher UVCV group and lower UVCV group (Table 1).

**Table 1.** The general clinical characteristics of the included participants.

	Total	UVSD		*p* Value	UVCV		*p* Value
		Higher (>=627.6)	Lower (<627.6)		Higher (>=0.29)	Lower (<0.29)	
No. of patients	283	142	141		149 (0.53)	134 (0.47)	
Age (years)	57.54 ± 14.52	55.18 ± 14.53	59.89 ± 14.19	0.006	59.07 ± 14.85	55.84 ± 14.01	.062
Male	151 (0.53)	89 (0.63)	62 (0.44)	0.001	76 (0.51)	75 (0.56)	.403
BMI (kg/m^2^)	22.70 ± 3.40	23.09 ± 3.65	22.30 ± 3.10	0.051	22.13 ± 3.17	23.33 ± 3.54	.003
Dialysis vintage (years)	3.38 (1.83–4.78)	3.23 (1.71–4.74)	3.45 (2.20–4.79)	0.404	3.02 (1.54–4.43)	3.46 (2.50–5.18)	.006
Comorbidities							
CVD	100 (0.29)	55 (0.39)	45 (0.32)	0.199	53 (0.36)	47 (0.35)	.931
Diabetes	81 (0.35)	43 (0.30)	38 (0.27)	0.488	37 (0.25)	44 (0.33)	.137
Vascular access				1			.038
Fistula/graft	274 (0.97)	137 (0.96)	137 (0.97)		141 (0.95)	133 (0.99)	
Catheter	9 (0.03)	5 (0.04)	4 (0.03)		8 (0.05)	1 (0.01)	
CKD etiology				0.534			.178
Glomerulonephritis	114 (0.40)	61 (0.43)	53 (0.38)		62 (0.42)	52 (0.39)	
Diabetes	73 (0.26)	39 (0.28)	34 (0.24)		32 (0.21)	41 (0.31)	
Hypertension	30 (0.11)	12 (0.08)	18 (0.13)		16 (0.11)	14 (0.10)	
Other	48 (0.07)	20 (0.14)	29 (0.21)		26 (0.17)	23 (0.17)	
Unknown	17 (0.17)	9 (0.06)	8 (0.06)		13 (0.09)	4 (0.03)	
IDH (%)	0.14 (0.07–0.22)	0.14 (0.08–0.24)	0.13 (0.07–0.21)	0.318	0.15 (0.08–0.28)	0.13 (0.07–0.19)	.005
24-hour urine volume (mL)	1000.0 (550.0–1500.0)	1000.0 (700.0–1500.0)	800.0 (500.0–1350.0)	0.147	1000.0 (700.0–1500.0)	800.0 (500.0–1187.5)	.149
Laboratory data							
Hemoglobin (g/L)	111.0 (96.3–120.75)	109.0 (95.0–119.8)	112.5 (98.3–122.0)	0.092	108.0 (95.0–120.0)	113.0 (99.5–121.0)	.171
Serum albumin (g/L)	39.70 (37.40–42.18)	39.80 (37.00–42.30)	39.60 (37.65–42.00)	0.948	39.75 (37.40–42.08)	39.65 (37.53–42.20)	.842
Serum Ca (mmol/L)	2.09 ± 1.88	2.09 ± 0.19	2.09 ± 0.19	0.975	2.09 ± 1.91	2.09 ± 0.19	.894
Serum P (mmol/L)	1.99 ± 0.56	2.06 ± 0.56	1.92 ± 0.54	0.031	1.98 ± 0.59	2.00 ± 0.52	.877
Serum iPTH (pg/mL)	468.9 (254.8–754.3)	521.8 (274.3–821.1)	363.0 (220.0–685.2)	0.019	492.0 (239.0–755.4)	450.3 (272.2–753.8)	.928
BNP^a^	575.0 (172.0–1535.0)	768.0 (167.5–1730.3)	461.0 (172.0–1304.5)	0.620	537.0 (204.0–1345.0)	634.0 (166.0–2300.0)	.592
UVSD	625.6 (528.2–755.3)	754.8 (681.9–873.6)	528.2 (461.1–593.3)	<0.001			
UVCV	0.29 (0.23–0.39)				0.39 (0.32–0.49)	0.23 (0.20–0.26)	<.001

UVSD: standard deviation of ultrafiltration volume variability; UVCV: coefficient of variation of ultrafiltration volume variability; BMI: body mass index; CVD: cardiovascular disease; CKD: chronic kidney disease; IDH: intradialytic hypotension; serum Ca: serum calcium; serum P: serum phosphorous; serum iPTH: serum intact parathyroid hormone; BNP: B-type natriuretic peptide.

^a^ BNP: only 152 patients were tested.

### UV variability and mortality

**Figure 3.**
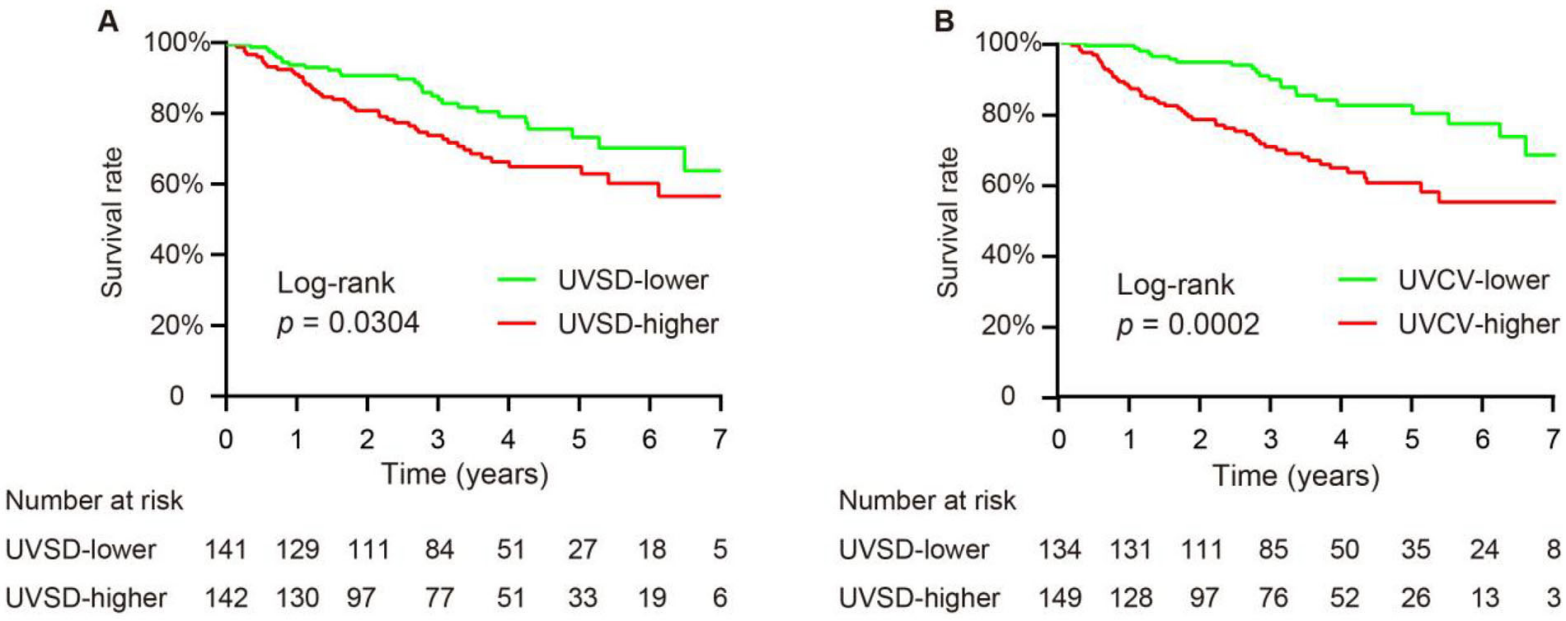
Survival comparing according to UVSD (A) or UVCV (B) between higher and lower groups using the Kaplan–Meier method.

**Figure 4.**
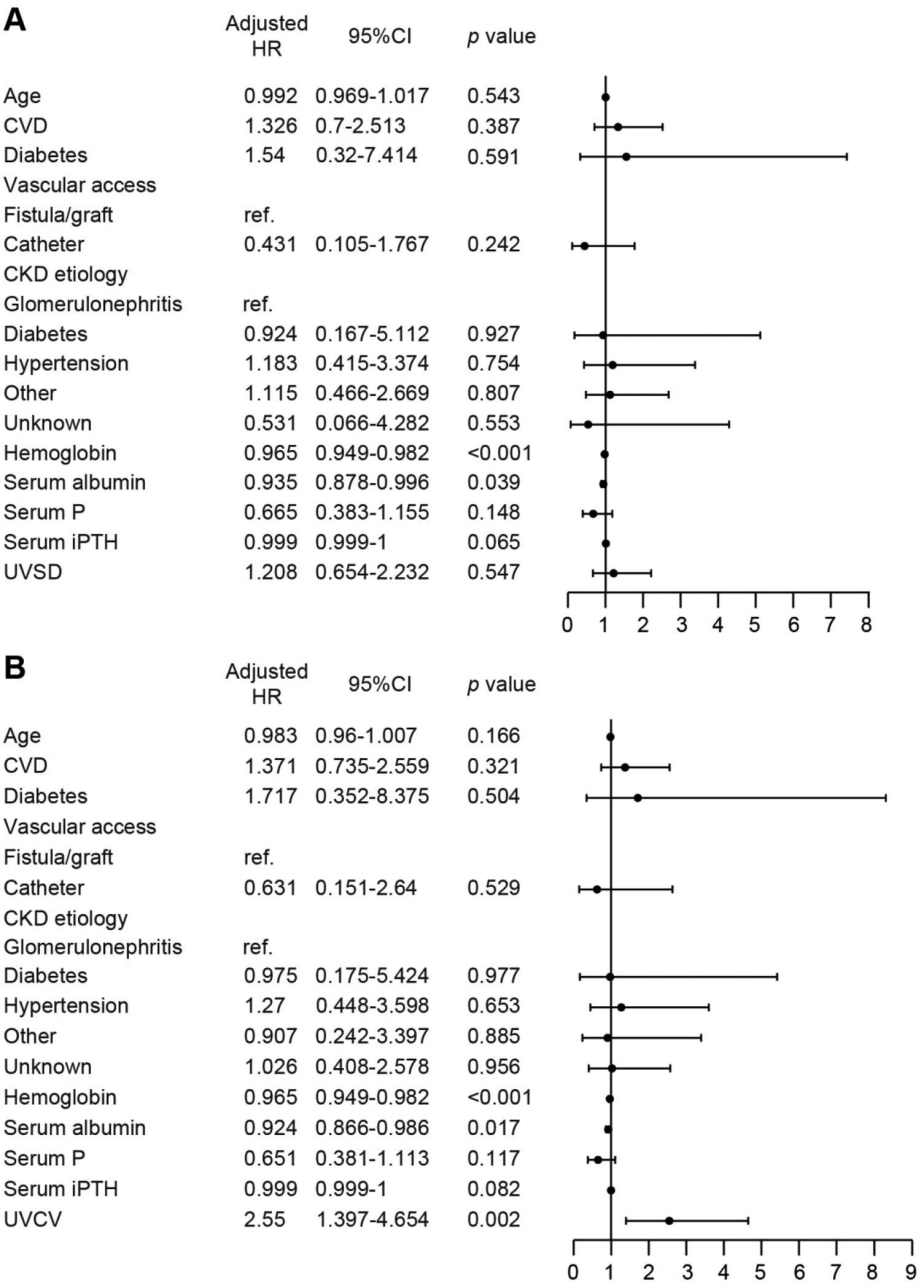
Multivariate analysis using the Cox proportional hazard regression analyses. All-cause mortality risk analysis according to the UVSD (A) or UVCV (B); adjusted for age, CVD, diabetes, vascular access, CKD etiology, hemoglobin, serum albumin, serum P, and serum iPTH.

During a median follow-up of 3.38 years (IQR 1.83–4.78), a total of 73 patients died. Next, we analyzed the potential risk factors for overall mortality. As determined by univariate Cox proportional hazard regression analyses, we found that age, hemoglobin, serum albumin, serum phosphorus, and serum iPTH were positively associated with all-cause mortality (Table 2). For categorical variables, we set non-CVD, nondiabetic, fistula/graft, and glomerulonephritis as references. We also found that the following variables were positively associated with all-cause mortality: CVD, diabetes, catheter use, diabetes, and CKD of unknown cause. The Kaplan–Meier curve illustrated that patients with higher UVSD or UVCV had a higher all-cause mortality (Figure 3). As a next step, we conducted a multivariable analysis to explore the association between UV variability and all-cause mortality. Cox proportional hazards models indicated that UVSD (HR 1.674 (95% CI 1.044–2.684), *p*=.030) and UVCV (HR 2.509 (95% CI 1.519–4.141), *p*<.001) (higher versus lower) were positively associated with all-cause mortality in univariate analyses, while after adjusting for age, CVD, diabetes, vascular access, CKD etiology, hemoglobin, serum albumin, serum phosphorus, and serum iPTH, only higher UVCV remained significantly associated with all-cause mortality in patients receiving HD (HR 2.55 (95% CI 1.397–4.654), *p*=.002) (Figure 4(A,B)).

**Table 2.** Univariate analysis of the associations between variables and all-cause mortality using Cox proportional hazard regression analyses.

Variables	HR	95% CI	*p* Value
Age	1.035	1.018–1.052	<.001
Male	0.899	0.567–1.424	.649
BMI	0.942	0.879–1.009	.089
CVD	1.992	1.243–3.192	.004
Diabetes	2.337	1.474–3.704	<.001
Vascular access	0.174	0.074–0.408	<.001
Fistula/graft	Ref.		
Catheter	5.748	2.453–13.471	<.001
CKD etiology			
Glomerulonephritis	Ref.		
Diabetes	3.696	2.026–6.743	<.001
Hypertension	2.14	0.915–5.006	.079
Other	1.965	0.945–4.088	.071
Unknown	3.201	1.065–9.625	.038
IDH	4.313	0.605–30.774	.155
24-hour urine volume	2.575	1.024–6.853	.156
Hemoglobin	0.950	0.937–0.962	<.001
Serum albumin	0.865	0.830–0.901	<.001
Serum Ca	2.021	0.574–7.124	.273
Serum P	0.457	0.277–0.755	.002
Serum iPTH	0.999	0.998–1.000	.004
UVSD	1.674	1.044–2.684	.030
UVCV	2.509	1.519–4.141	<.001

HR: hazard ratio; SD: standard deviation.

### Subgroup analysis

**Figure 5.**
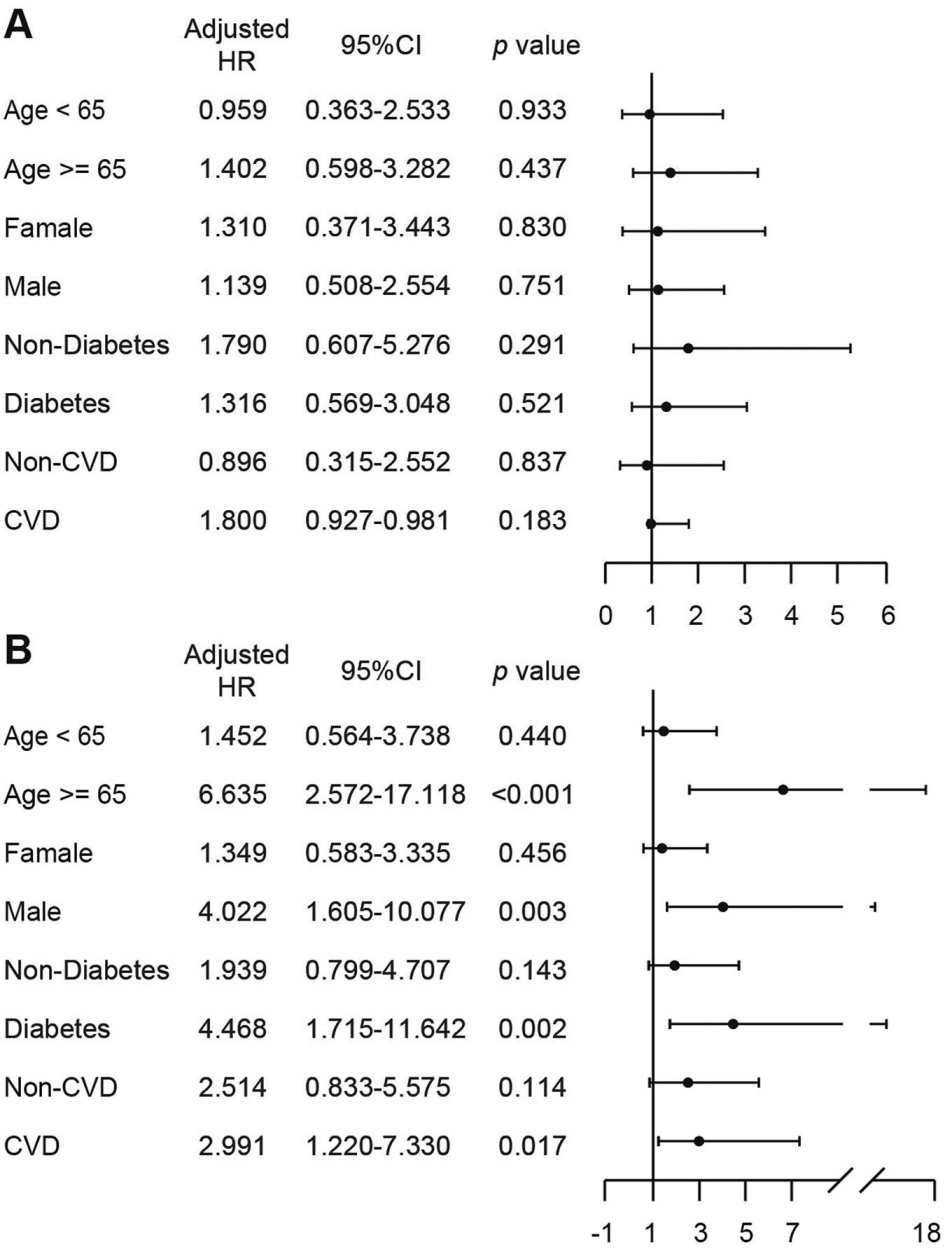
Subgroup analysis according to age, gender, with or without comorbidities. All-cause mortality risk analysis according to UVSD (A) or UVCV (B); adjusted for age, CVD, diabetes, vascular access, CKD etiology, hemoglobin, serum albumin, serum P, and serum iPTH.

Further subgroup analyses were conducted to determine the relationship between UV variability and all-cause mortality in specific populations. Patients were grouped by age, sex, and comorbidities. Similar to the analysis for the whole population (Figure 5(A)), UVSD was not a useful predictor of all-cause mortality. Interestingly, we found that UVCV had better predictive ability in patients who were older (HR 6.635 (95% CI 2.572–17.118), *p*<.001), male (HR 4.022 (95% CI 1.605–10.077), *p*=.003), had diabetes (HR 4.468 (95% CI 1.715–11.642), *p*=.002) or had CVD (HR 2.991 (95% CI 1.220–7.330), *p*=.017) than in the entire population (Figure 5(B)).

### Survival rates for UV variability

**Figure 6.**
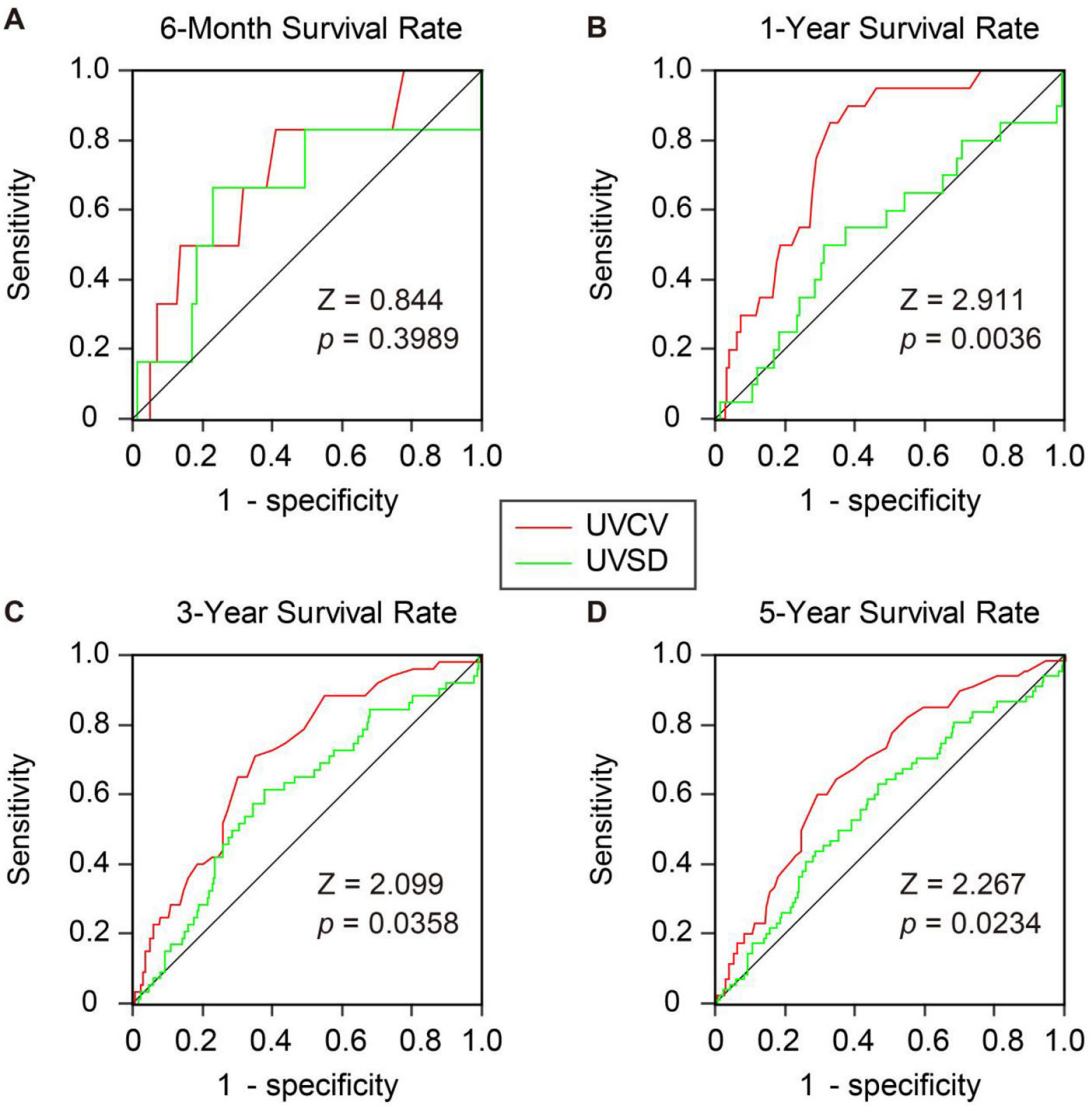
Comparison of the area under the curve (AUC) between UVSD (green) and UVCV (red) in predicting 6-month (A), 1-year (B), 3-year (C) and 5-year (D) survival rate. The significances of ROC AUC between UVCV and UVSD were tested by *Z* test.

Finally, we compared UVSD and UVCV in predicting survival rates at different time points. ROC analysis was used to compare the predictive efficiency of two UV variability indices. After applying Bonferroni’s correction for multiple testing, our results showed that the ROC areas for UVSD and UVCV were comparable at six months (*p*=.3989), 3 years (*p*=.0358), and 5 years (*p*=.0234). Only at 1 year, UVCV had a better predictive ability for long-term survival than UVSD (*p*=.0036) (Figure 6).

## Discussion

The present study demonstrates that visit-to-visit UV variability could predict all-cause mortality in patients receiving HD. Compared with UVSD, UVCV might be a better predictor for long-term survival, especially in patients who are older, male, and those with comorbidities.

To the best of our knowledge, this study is the first to investigate the association between visit-to-visit UV variability and all-cause mortality in patients with HD. Although the underlying causes were not clear, our data allowed for some speculations. Volume overload is prevalent among HD patients because kidneys are unable to maintain fluid homeostasis in ESRD patients [15]. Kidneys are subjected to fluid accumulation during interdialytic intervals. However, excessive interdialytic weight gain (IDWG) and the consequent excessive intradialytic weight loss constitute a cyclical multiple-system organ stress, especially in the cardiovascular system [16]. Previous studies have shown that higher IDWG results in cardiac chamber remodeling [17], diastolic dysfunction and myocardial stunning [18]. These cardiovascular injuries are associated with increased all-cause and cardiovascular mortality [19–21]. These results indicate that a large fluctuation in volume status before and after HD predicts a poor prognosis. In the present study, visit-to-visit UV variability reflects another fluctuation of volume status. This fluctuation derives from dialysis sessions, distinct from that before and after HD.

Specifically, lower visit-to-visit UV variability means more stable fluid and solute removal between dialysis sessions, whether high or low absolute UV, which is theoretically helpful for the body to establish a balance of volume. This is of vital importance for maintaining cardiovascular function. Patients with higher UV variability are more vulnerable to experiencing large fluctuations in fluid and solutes between dialysis sessions. This poor condition goes against the body’s requirement of maintaining proper multiple-system organ function. For the cardiovascular system, higher UV variability increases the incidence of hypotension caused by excessive ultrafiltration or heart failure caused by insufficient ultrafiltration. Therefore, increased UV variability accompanies an increased risk of cardiovascular function impairment and poor outcomes. From this perspective, the link between higher UV variability and increased all-cause mortality seems reasonable and plausible.

Moreover, other variables’ variability among HD patients has also been studied. These results similarly show that high variability was associated with poor outcomes. For instance, numerous studies have shown that higher blood pressure variability is a risk factor for short- and long-term cardiovascular and all-cause mortality [22–26]. Additionally, several other studies reported that higher variabilities of serum phosphorus [27], serum albumin [28], hemoglobin [29], and heart rate [30] could also predict mortality in HD patients. Our findings of higher UV variability, together with the above results, support that higher variability is associated with poor clinical outcomes, although the reasons behind these associations are unclear and need further exploration.

In addition, many studies investigating risk factors for mortality in the HD population have revealed that patients who are male and older, with preexisting CVD and DM had a higher risk of cardiac death [31–34], indicating that these populations were more susceptible to cardiovascular disease. Our results also revealed that higher UV variability corresponds to higher risk in older, male, and comorbid populations (Figure 5). This may be attributed to the preexistence of or predisposition to cardiovascular diseases of such populations.

There are several limitations of the present study, not merely due to the observational nature of the study design. First, the sample size of our study was relatively small. Second, the follow-up of the study was too short to predict accurate long-term survival. Finally, the outcome was only all-cause mortality without cardiovascular mortality or other outcomes because of missing data.

## Conclusions

Our study demonstrates that higher UV variability is associated with increased all-cause mortality in patients receiving HD. This predictive effect is more accurate among older and male patients with comorbidities. The benefit of maintaining stable UV to improve clinical outcomes warrants further studies.

## Data Availability

The data that support the findings of this study are not publicly available due to their containing information that could compromise the privacy of research participants but are available from the corresponding author.
